# Targeting Glutamine Metabolism Ameliorates Autoimmune Hepatitis *via* Inhibiting T Cell Activation and Differentiation

**DOI:** 10.3389/fimmu.2022.880262

**Published:** 2022-05-19

**Authors:** Qiang Yu, Honghu Tu, Xueyi Yin, Chang Peng, Chuanyun Dou, Wenhua Yang, Wenbiao Wu, Xiaotong Guan, Jia Li, Hexin Yan, Yi Zang, Haowen Jiang, Qiang Xia

**Affiliations:** ^1^Department of Liver Surgery, Renji Hospital, School of Medicine, Shanghai Jiao Tong University, Shanghai, China; ^2^School of Chinese Materia Medica, Nanjing University of Chinese Medicine, Nanjing, China; ^3^State Key Laboratory of Drug Research, Shanghai Institute of Materia Medica, Chinese Academy of Sciences, Shanghai, China; ^4^School of Pharmacy, University of Chinese Academy of Sciences, Beijing, China; ^5^School of Pharmaceutical Science and Technology, Hangzhou Institute for Advanced Study, University of Chinese Academy of Sciences (UCAS), Hangzhou, China; ^6^Department of Anesthesia, Renji Hospital, School of Medicine, Shanghai Jiao Tong University, Shanghai, China; ^7^Shanghai Engineering Research Center of Transplantation and Immunology, Shanghai, China; ^8^Shanghai Institute of Transplantation, Shanghai, China

**Keywords:** glutamine metabolism, autoimmune hepatitis (AIH), T cells activation and differentiation, mTOR signaling, SLC7A5

## Abstract

**Background:**

Autoimmune hepatitis (AIH) is mediated by a cascade of T cell-mediated events directed at liver cells and persistent inflammation within the liver can eventually result in liver cirrhosis. Targeting glutamine metabolism has an impact on T cell activation and differentiation. However, the effect of glutamine metabolism blocking upon AIH remains unknown. We use glutaminase antagonist 6-diazo-5-oxo-L-norleucine (DON) for *in vitro* assays and its prodrug 2-(2-amino-4-methylpentanamido)-DON (JHU083) for *in vivo* assays to investigate the potential therapeutic effect and molecular mechanism of glutamine metabolism blocking in an AIH murine model.

**Methods:**

AIH mice were treated with JHU083 or vehicle before concanavalin A (ConA) administration, and disease severity was examined. Then activation and differentiation [including Th1/Th17 cells and cytotoxic T lymphocytes (CTL)] of T cells from Vehicle-WT, JHU083-AIH and Vehicle-AIH mice were tested. Furthermore, *in vitro* T cell activation and differentiation were measured using separated splenocytes stimulated with ConA with or without DON. The activation and differentiation of T cells were tested using flow cytometry, qRT-PCR and ELISA. Phosphorylation level of mammalian target of rapamycin (mTOR) and 70 kDa ribosomal protein S6 kinase (P70S6K) were examined by western blotting.

**Results:**

JHU083 and DON significantly suppressed the activation of T cells and inhibited the differentiation of Th1/Th17 cells and CTL *in vivo* and *in vitro*. Besides, we demonstrated that glutamine metabolism blocking inhibited T cells activation and differentiation through decreasing the mRNA expression of amino acid transporter solute carrier family 7 member 5 (SLC7A5) and mitigating the activation of mTOR signaling.

**Conclusions:**

We proved that targeting glutamine metabolism represents a potential new treatment strategy for patients with AIH and other T cell-mediated disease. Mechanistically, we demonstrated that glutamine metabolism blocking inhibits T cells activation and suppresses the differentiation of Th1/Th17 cells and CTL.

## Introduction

Autoimmune hepatitis (AIH) is a self-perpetuating inflammatory liver disease, and the misdiagnosis or delayed treatment of AIH can lead to liver cirrhosis, liver cancer, liver transplantation and even rapid death (1–3). AIH occurs globally in all ethnicities and affects children and adults of all ages, with a female predominance (4). It is reported that the point prevalence is up to be 10-25 per 100,000 population in the European region, and 5-25 per 100,000 population in the Asia-Pacific region (5). Although the specific pathogenesis of AIH is still unclear, it is indicated that the liver injury of AIH is mediated by a cascade of T cell-mediated events directed at liver cells and persistent inflammation within the liver can eventually result in liver cirrhosis (6).

The precise etiology and pathophysiology of AIH remains largely unknown, and lacking valid animal models for AIH research makes this situation a vicious circle. Thus, related basic research in this field is relatively limited compared with other types of hepatitis. However, some animal models can partially represent the pathogenesis of AIH, among which, intravenous injection of concanavalin A (ConA) is widely used in AIH research and this model shows the specific participation of T cells in promoting liver injury (7). ConA promotes T cells activation and differentiation into effector cells, followed by the secretion of pro-inflammatory cytokines and immune-mediated damage of liver cells caused by the cytokines and cascade reaction (8–10). The ConA-induced AIH model is considered as a valuable method for investigating the specific mechanism underlying T cell activation and differentiation in AIH.

Glutamine is a conditionally essential amino acid in rapidly proliferating cells and glutaminolysis is a very important source of energy for effector T cells (11). Glutaminase (GLS) is the first enzyme of glutaminolysis and converts glutamine to glutamate, which can be used in protein synthesis and glutathione generation (12). Early studies show that glutamine metabolism blocking not only diminishes T cells activation (13, 14), but also decreases the differentiation of T cells (15–17). 6-diazo-5-oxo-L-norleucine (DON) is a glutamine analog that competitively inhibits glutamine metabolism and suppresses the activation and differentiation of T cells (18–20). Thus, we aim to investigate the effect of glutamine metabolism blocking on ConA-induced AIH model. Despite the efficacy of DON in basic researches, its substantial peripheral toxicity has hampered its approval as a chemotherapeutic agent (21, 22). We use ethyl 2-(2-amino-4-methylpentanamido)-DON (JHU083), an orally available prodrug form of DON developed by Atsushi Kamiya and coworkers (23) to control oral bioavailability and decrease toxicity, for *in vivo* assays.

Because the mTOR signaling cascade plays a central role in the regulation of T cell activation and differentiation (24–27), blocking glutamine metabolism may affect the activation of mTOR signaling so as to influence T cells activation and differentiation. In this study, we aim to investigated whether targeting glutamine metabolism inhibits activation and differentiation of T cells and ameliorates the progression of ConA-induced AIH model.

## Materials and Methods

### Animal Experimental Design

C57/BL6 mice (female, 6- to 7-week-old) were purchased from Shanghai SLAC Laboratory Animal Co (Shanghai, China). All animal procedures were performed in accordance with the Guidelines for Care and Use of Laboratory Animals of Shanghai Institute of Materia Medica (SIMM), Chinese Academy of Sciences and approved by the Institutional Animal Care and Use Committee of SIMM.

Mice were randomly divided into three groups: wild-type (WT) group (n=4, Vehicle-WT), vehicle group (Vehicle-AIH, n=6) and JHU083-treated group (JUH083-AIH, n=6). Induction of AIH was based on the treatment of mice with ConA (8mg/kg body weight (BW), C2010, Sigma-Aldrich, USA). Mice in Vehicle-WT group were treated with 5% methyl cellulose (MC) dissolved in saline by oral gavage 24h and 1h before administration of saline *via* intravenous injection. The Vehicle-AIH group received administration of 5% MC by oral 24h and 1h before ConA injection, while mice in JUH083-AIH group were treated with JHU083 (0.3mg/kg BW, HY-122218, MedChemExpress, NJ, USA) dissolved in 5% MC *via* gavage 24h and 1h before ConA treatment. Blood and liver tissues were collected from mice that were sacrificed 6h after ConA treatment. Serum alanine aminotransferase (ALT), aspartate aminotransferase (AST), lactate dehydrogenase (LDH) and alkaline phosphatase (ALP) were analyzed using an automatic biochemical analyzer (AU480, Beckman Coulter, Pasadena, CA, USA) to assess liver injury. For survival analysis, concentration of ConA was adjusted to a lethal dose (20mg/kg) and mice were under observation until 48h after ConA injection.

### Histological Staining and TUNEL Assay

The tissues were fixed in 4% paraformaldehyde and embedded in paraffin. Tissue sections were prepared and stained with hematoxylin-eosin (H&E). Terminal Deoxynucleotidyl Transferase (TdT)-mediated dUTP Nick-End Labeling (TUNEL) staining was performed in paraffin-embedded liver sections using the TUNEL Kit (C1090, Beyotime, Haimen, China). Finally, the slides were scanned using a fluorescence microscope (1013688, PE Vectra3, Perkin Elmer, Waltham, MA, USA).

### Isolation of Splenocytes and Cell Culture

Spleens were dissected from C57BL/6 mice and cell suspensions were prepared as following steps. Spleens were milled and centrifuged at 400g for 5 minutes. Red blood cells were lysed and splenocytes were washed with 10ml phosphate buffer solution (PBS). Cells were resuspended with complete medium (RPMI-1640 medium supplemented with 10% inactivated fetal bovine serum, 1% penicillin-streptomycin solution, 10mM 4-(2-hydroxyethyl)-1-piperazineethanesulfonic acid (HEPES), 1mM glutamine, 50μM β-mercaptoethanol and 1mM sodium pyruvate) for further culture or for cytometric assays.

### T Cell Activation and Differentiation

Isolated splenocytes were activated with ConA (1.5μg/ml) with or without 2.5μM DON (HY-108357, MedChemExpress, NJ, USA). After 24h, the expression of CD69 and CD25 were analyzed by flow cytometry to evaluate the activation of CD4(+) and CD8(+) T cells. For T cell differentiation analysis, splenocytes were stimulated using ConA (1.5μg/ml) with or without DON (2.5μM). GlogiPlug (555028, BD Biosciences, USA) was added 18h after for the next 24 h of incubation. The cells were first stained with monoclonal antibodies CD4 and CD8 for 20 min at room temperature, then fixed using a Fixation/Permeabilization Solution Kit (555028, BD Biosciences, USA), and finally stained with IFN-γ, IL4, IL17 and Granzyme B antibodies. Flow cytometry analysis was conducted by flowJo_V10 software (BD Biosciences, USA). For *in vivo* T cells activation and differentiation tests, splenocytes were separated from mice in WT-Vehicle, Vehicle-AIH and JHU083-AIH group and stained with above antibodies for flow cytometry analysis. The antibodies used in the flow cytometry were as follows: CD4 (FITC, E-AB-F1097C), CD8a (PE, E-AB-F1104D), CD25 (PE-Cy5, E-AB-F1102G), CD69 (PE-Cy7, E-AB-F1187H), IL4 (PE, E-AB-F1204UD), IL17A (APC, E-AB-F1272E) were purchased from Elabscience Biotechnology (Wuhan, China), antibodies IFNγ (APC-Cy7, cat# 561479) was purchased from BD Biosciences (UK), and Granzyme B (PE-Cy7, cat# 372213) was purchased from Biolegend (San Diego, CA, USA). SLC7A5 antagonist LAT1-IN1 (HY-108540) was purchased from MedChemExpress (NJ, USA).

### Enzyme Linked Immunosorbent Assay

The IFN-γ Mouse Uncoated ELISA Kit (EK280/3-48, Multi Science, China) and IL17 Mouse Uncoated ELISA Kit (EK217/2-48, Multi Science, China) were used to measure the levels of IFN-γ and IL17 in the serums of mice in Vehicle-WT, Vehicle-AIH and JHU083-AIH group and the supernatants of unstimulated splenocytes or splenocytes that were stimulated with ConA in the presence of vehicle or DON. Briefly, the serums and supernatants were collected and added to the well of corresponding microplates for 2h. After the samples were washed with wash solution, 100μL of mouse IFN-γ or IL17 conjugate was added to each well for 2h. The washing process was repeated, and 200μL of Substrate Solution was added and incubated for 30 min. Then, 50μL of Stop Solution was added to each well, and the optical density was determined within 30 min using a microplate reader (SpectraMax M5, Molecular Devices, Santa Clara, CA, USA) set to 450nm.

### Western Blotting

Protein samples were homogenized with SDS loading buffer and boiled to denature the proteins. Samples with different sizes were separated by 10% SDS-polyacrylamide gel electrophoresis, and transferred to NC membranes. After blocking with 5% non-fat milk solution for 1h, membranes were incubated with β-Actin, P-mTOR, mTOR, P-P70S6K P70S6K at 4°C overnight. Then, membranes were incubated with HRP-linked anti-rabbit IgG (7074, Cell Signaling Technology, Trask Lane Danvers, MA, USA) at room temperature for 1h. Finally, the membranes were stained with ECL detection buffer (1705061, Bio-Rad, Hercules, CA, USA) and visualized using ChemiDoc system (Bio-Rad, Hercules, CA, USA). Primary antibodies including β-Actin (12620), P-mTOR (2971S), mTOR (2972S), P-P70S6K (97596), P70S6K (34475) were purchased from Cell Signaling Technology (Trask Lane Danvers, MA, USA).

### Quantitative Real-Time PCR

Total RNA was extracted using TRIzol reagent (9109, TaKaRa, Dalian, China) followed by cDNA synthesis using RT Master Mix (RR036B, TaKaRa, Dalian, China). Subsequently, cDNA was used to measure the mRNA levels of IFN-γ, IL4, IL17, SLC7A5 and SLC1A5 using a Real-Time PCR System (Roche, Basel, Switzerland). β-Actin was used as the normalization control. All reactions were performed in triplicates. The relative quantifications were measured by the comparative CT method. The primer sequences used were as follows: IFN-γ: forward: CAGCAACAGCAAGGCGAAA, reverse: CTGGACCTGTGGGTTGTTGAC; IL-4: forward: CGCCATGCACGGAGATG, reverse: CGAGCTCACTCTCTGTGGTGTT; IL17: forward: ATCTGTGTCTCTGATGCTGTTGCTG, reverse: TGGAACGGTTGAGGTAGTCTGAGG; SLC7A5: forward: CTGGATCGAGCTGCTCATC, reverse: GTTCACAGCTGTGAGGAGC; SLC1A5: forward: ATCGCACAACTAAACGGGGT, reverse: ACTGCTTCCAGGATGATGGC; β-Actin: forward: AACAGTCCGCCTAGAAGCAC, reverse: CGTTGACATCCGTAAAGACC.

### Statistical Analysis

Experimental data were expressed as the mean ± standard error of mean (SEM), and comparisons between two groups were performed using one-way ANOVA analysis. Data were analyzed using GraphPad Prism 8.0.2. Statistical significance was determined at *P* < 0.05 (*); *P* < 0.01 (**); and *P* < 0.001 (***).

## Results

### JHU083 Attenuates ConA-Induced AIH in Mice

**Figure 1:**
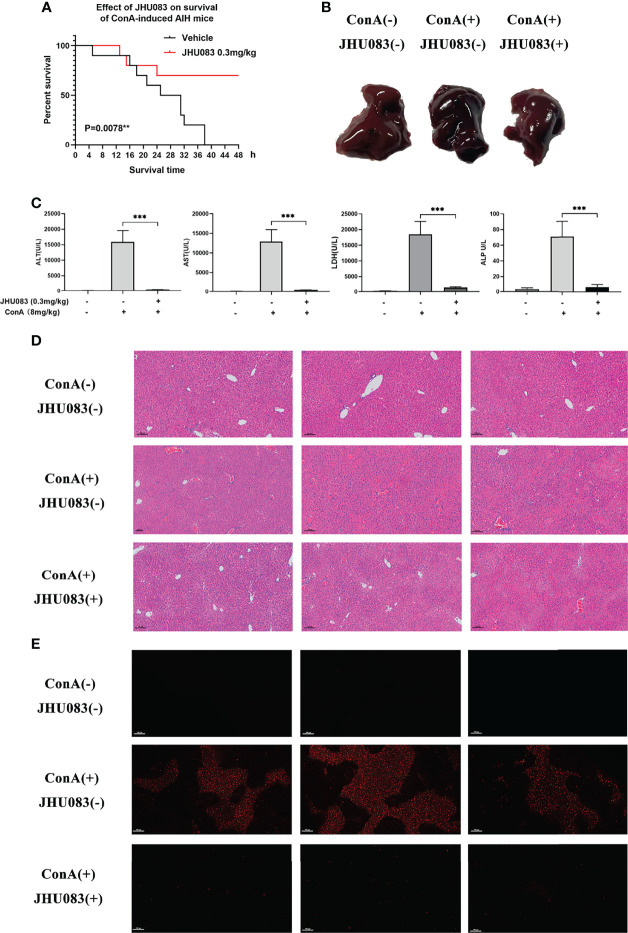
JHU083 attenuated ConA-induced AIH. **(A)**. Survival of 20ml/kg ConA-induced autoimmune hepatitis mice gavaged with vehicle or 0.3mg/kg JHU083 24h and 1h before ConA injection. n = 10. **(B–E)** Mice of Vehicle-AIH and JHU083-AIH group were intravenously injected with 8 mg/kg ConA, and mice of Vehicle-WT group were injected with normal saline of equal volume. Mice of JHU083 group were gavaged with 0.3mg/kg JHU083 24h and 1h before ConA injection, and WT and vehicle group were treated with equal vehicle at the same time points. Representative macroscopic images of livers **(B)**, quantification of serum ALT, AST, LDH and ALP **(C)**, H&E staining **(D)** and TUNEL assay **(E)** were presented 6h after ConA injection. The scale bar for H&E staining and TUNEL assay is 100μm. Red fluorescence in TUNEL assay indicates positive signals of apoptosis. Data were expressed as means ± SEM. **P <0.01 and ***P <0.001.

In order to verify the hepatoprotective effect of glutaminase antagonist JHU083 in a ConA-induced AIH model, the survival times of vehicle and JHU083 (0.3mg/kg BW) (28) treated mice in this mice model were investigated. Vehicle-AIH and JHU083-AIH mice were intravenously injected with a high dose of ConA (20mg/kg BW). Vehicle-AIH mice began to die 4h after injection and all mice died within 38h, while survival rate of JHU083-AIH mice was 70% at 24h after ConA injection and did not change after extension to 48h. On the basis of the survival curve, it was proved that JHU083 treatment greatly reduced the death rate of ConA-induced AIH mice (**Figure 1A**). Although AIH mice were ameliorated after treatment with JHU083 under the induction of 20mg/kg ConA (**Supplementary Figure 1**), the pathological damages under such ConA concentration would be too severe. Therefore, we chose a non-lethal dosage instead to compare the alteration of transaminase, conditions of cells and pathological damages. Then, ConA was adjusted to a lower dose (8mg/kg), and blood and liver specimens were collected 6h post ConA injection. In JHU083-AIH group, liver congestion and necrosis were obviously mitigated in a macroscopic level (**Figure 1B**) and serum ALT, AST, LDH and ALP (**Figure 1C**) were significantly lower than mice in Vehicle-AIH group. Liver specimens were stained with H&E. At 6h after ConA administration, marked liver hyperemia and necrosis of liver cells were observed in vehicle mice rather than in JHU083 treated mice (**Figure 1D**). No pathological injury was found in Vehicle-WT mice. Apart from these, the TUNEL assay indicated that TUNEL-positive cells decreased significantly after JHU083 treatment (**Figure 1E**), indicating decreased apoptosis of liver cells. These results proved that blocking glutamine metabolism ameliorated the progression of ConA-induced AIH in mice.

### JHU083 Inhibited the Activation and Differentiation of T Cells *In Vivo*

**Figure 2:**
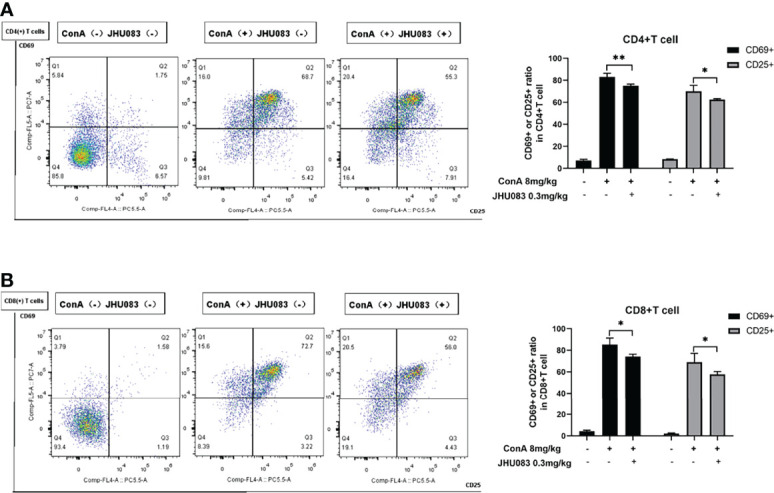
JHU083 inhibited T cells activation *in vivo*. Mice of Vehicle-AIH and JHU083-AIH group were intravenously injected with 8ml/kg ConA, and Vehicle-WT group was injected with normal saline of equal volume. JHU083-AIH group was gavaged with 0.3mg/kg JHU083 24h and 1h before ConA injection, and mice of Vehicle-WT and Vehicle-AIH group were treated with equal vehicle at the same time points. **(A, B)** Flow cytometry was applied to analyze the expression of CD25 and CD69 of CD4(+) **(A)** and CD8(+) **(B)** T cells. Data were expressed as means ± SEM. *P < 0.05 and **P <0.01.

According to Ruoning Wang (29), we transplanted CFSE-labeled splenocytes into mice before ConA injection. After 6h ConA stimulation, there was no proliferation in the transplanted splenocytes (**Supplementary Figure 2**). Thus, we focused on the activation and differentiation of T cells. Given that activated effector T cells need glutamine metabolism to provide energy (11), we first measured the activation of T cells upon glutamine metabolism blocking *in vivo*. CD25 and CD69 are two markers expressed on the surface of activated T cells (30). To investigate the effect of JHU083 on the activation of T cells in AIH mice, we evaluated the activation of CD4(+) and CD8(+) T cells in spleens extracted from mice in WT-Vehicle, Vehicle-AIH and JHU083-AIH group *via* the proportions of CD25 or CD69 positive cells. Consistent with our findings on pathology, JHU083 significantly reduced percentages of activated CD4(+) and CD8(+) T cells (**Figures 2A, B**).

**Figure 3:**
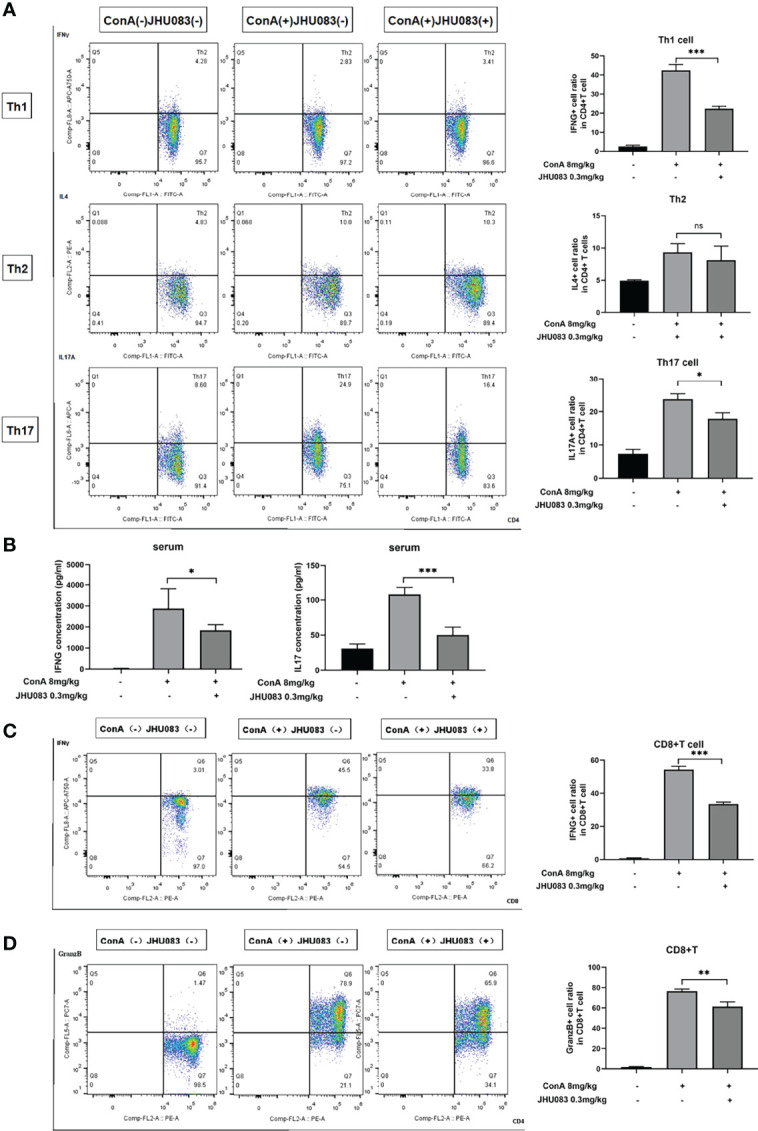
JHU083 inhibited T cells differentiation *in vivo*. Mice of Vehicle-AIH and JHU083-AIH group were intravenously injected with 8ml/kg ConA, and Vehicle-WT group was injected with normal saline of equal volume. JHU083-AIH group was gavaged with 0.3mg/kg JHU083 24h and 1h before ConA injection, and Vehicle-WT and Vehicle-AIH group were treated with equal vehicle at the same time points. **(A)** Flow cytometry was applied to analyze the Th1 cell percentage *via* IFNγ expression, the Th2 cell percentage *via* IL4 expression, and the Th17 cell percentage *via* IL17A expression. **(B)** ELISA was applied to analyze serum IFNγ (Th1 cells) and IL17 (Th17 cells) secretions. Flow cytometry was also applied to analyze the differentiation of CD8(+) T cells into cytotoxic T lymphocytes, evidenced by secreting IFNγ **(C)** and Granzyme **(D)**. Data were expressed as means ± SEM. *P < 0.05, **P <0.01, and ***P <0.001. ns, not significant.

To further explore the effect of JHU083 on the differentiation of CD4(+) and CD8(+) T cells in AIH mice, the differentiation marker of CD4(+) (IFNγ for Th1 cells, IL4 for Th2 cells and IL17A for Th17 cells) and CD8(+) T cells (IFNγ and Granzyme B for CTLs) were evaluated using flow cytometry. We observed that the frequency of IFNγ- and IL17A-expressing CD4(+) T cells instead of IL4-expressing CD4(+) T cells were significantly lower in the spleens of JHU083-AIH mice compared to that of Vehicle-AIH mice (**Figure 3A**). Lower IFNγ and IL17 levels were presented in the serums of JHU083-AIH mice than in Vehicle-AIH mice (**Figure 3B**). Besides, the frequency of CD8(+) T cells expressing IFNγ (**Figure 3C**) or Granzyme B (**Figure 3D**) was significantly reduced after JHU083 treatment. The above outcomes indicated that blocking glutamine metabolism had negative effects on activation and differentiation of T cells *in vivo*.

### DON Treatment Suppressed the Activation and Differentiation of T Cells *In Vitro*

**Figure 4:**
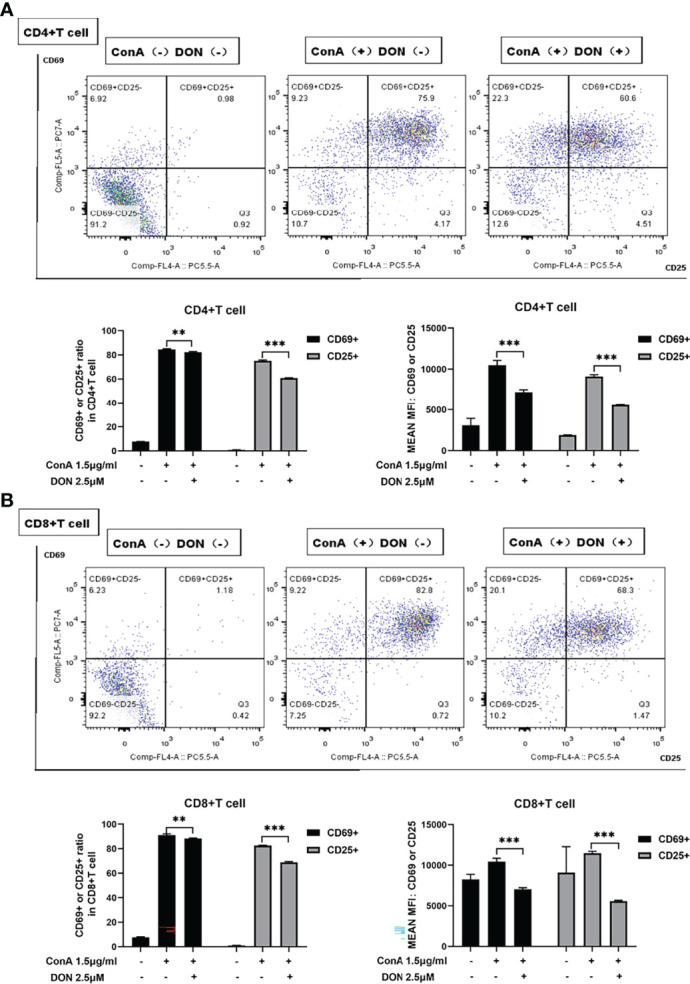
DON treatment suppressed T cells activation *in vitro*. Freshly separated spleen cells were stimulated with 1.5mg/ml ConA with or without treating 2.5μM DON, and cells were analyzed 24h after ConA stimulation. **(A, B)** The expressions of activation markers CD25 and CD69 in CD4(+) **(A)** and CD8(+) **(B)** T cells were examined by flow cytometry. Data were expressed as means ± SEM. **P <0.01, and ***P <0.001.

Consistent with the results *in vivo*, ConA did not induce proliferation within 24h *in vitro* (**Supplementary Figure 3**). To evaluate the effects of blocking glutamine metabolism on T cells *in vitro*, we used DON to substitute JHU083 to investigate whether DON might suppress the activation of T cells. Splenocytes of mice were separated and cultured in RPIM 1640 medium and activated by 1.5μg/ml ConA in the presence or absence of DON (2.5μM) (**Supplementary Figure 4**). 24h after activation, splenocytes were collected and stained with CD4, CD8 and activation markers CD25 and CD69, and the proportions of CD25(+) or CD69(+) cells and median fluorescence intensity (MFI) of CD25(+) or CD69(+) cells were analyzed. Although CD25 can be also expressed on regulatory T cells, we assessed the proportion of CD4+Foxp3+ Treg cells and found that DON did not affect the proportion of Treg cells (**Supplementary Figure 5**). As shown in **Figures 4A, 4B**, splenocytes treated with DON had lower expressions of CD25 and CD69 in CD4(+) and CD8(+) T cells.

**Figure 5:**
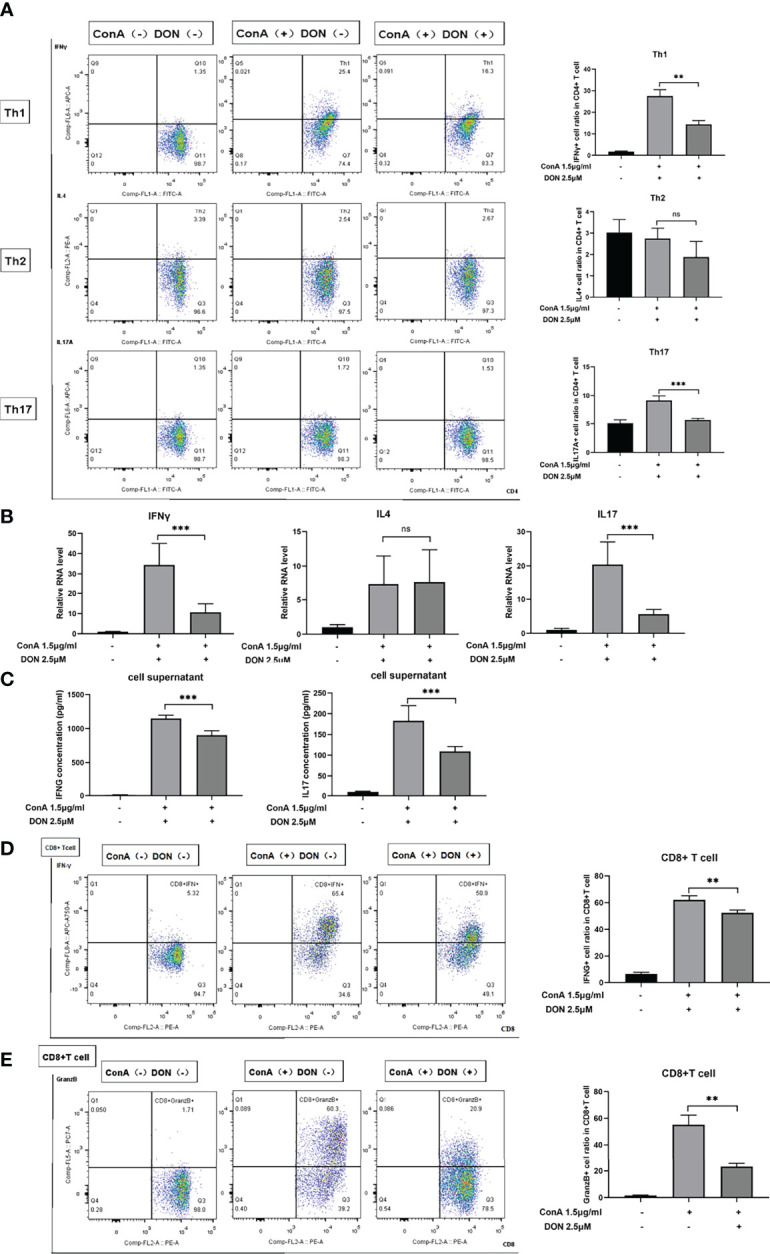
DON treatment suppressed T cells differentiation *in vitro*. Freshly separated spleen cells were stimulated with 1.5mg/ml ConA with or without treating 2.5μM DON, and cells were analyzed 24h after ConA stimulation. **(A)** The differentiation markers of CD4+ T cells (IFNγ for Th1 cells, IL4 for Th2 cells and IL17 for Th17 cells) were detected using flowcytometry. **(B)** qRT-PCR was applied to analyze IFN-γ, IL4 and IL17 mRNA levels. **(C)** ELISA was applied to analyze supernatant IFN-γ and IL17 secretions. Flow cytometry was also applied to detect the production of activation markers of CTL, such as IFNγ **(D)** and Granzyme B **(E)**. Data were expressed as means ± SEM. **P <0.01 and ***P <0.001, ns, not significant.

To further characterize the effect of DON on the differentiation of CD4(+) and CD8(+) T cells, the above-mentioned differentiation markers of CD4(+) and CD8(+) T cells were tested. DON significantly inhibited the differentiation of Th1 and Th17 cells rather than Th2 cells (**Figure 5A**). Consistently, DON reduced mRNA expression (**Figure 5B**) and production (tested by ELISA, **Figure 5C**) of IFNγ and IL17. In addition, we proved that DON suppressed CD8(+) T cells to turn into CTL, which was evidenced by lower productions of IFNγ- (**Figure 5D**) and Granzyme B-expressing T cells (**Figure 5E**). Thus, GLS blocking played a critical role in inhibiting T cells activation and differentiation *in vitro*.

### The Effects of Blocking Glutamine Metabolism on Activation and Differentiation of T Cells Were Mediated by mTOR Signaling and Amino Transporter SLC7A5

Mammalian target of rapamycin (mTOR) signaling is a master regulator of cell metabolism and promotes anabolic processes such as protein synthesis (31). The activation of mTOR signaling is dependent on growth factors and nutrients in cancer cells (32). For instance, leucine can promote mTOR signaling activation *via* binding to sestrin2 and arginine binds to CASTOR1 (33). Glutamine activates mTOR signaling in a RAG-dependent manner *via* glutaminolysis (34). In addition, many studies have noted that the activation and differentiation of T cell are strongly correlated with mTOR signaling (24–27). We hypothesized that blocking glutamine metabolism may affect mTOR signaling in T cells in an amino acid dependent way.

**Figure 6:**
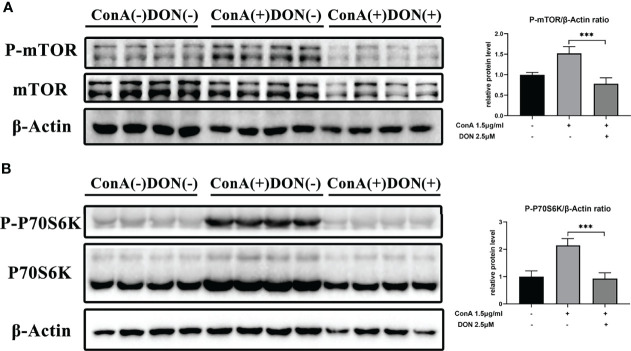
The inhibition of activation and differentiation of T cells was mediated by mTOR signaling. Freshly separated spleen cells were stimulated with 1.5 mg/ml ConA with or without treating 2.5μM DON, and cells were collected and proteins were extracted 24h after ConA stimulation. Western blotting was applied to analyze the levels of phosphorylated mTOR **(A)** and phosphorylated P70S6K **(B)**. Data were expressed as means ± SEM. ***P <0.001.

Thus, we examined the phosphorylated protein levels of mTOR signaling using proteins extracted from splenocytes that were activated by 1.5μg/ml ConA with or without treating 2.5μM DON for 24h. After activating by ConA, the protein levels of phosphorylated mTOR and 70 kDa ribosomal protein S6 kinase (P70S6K) increased sharply. But in the presence of DON, the levels of phosphorylated mTOR and P70S6K instead of their unphosphorylated proteins significantly decreased (**Figures 6A, B**).

**Figure 7:**
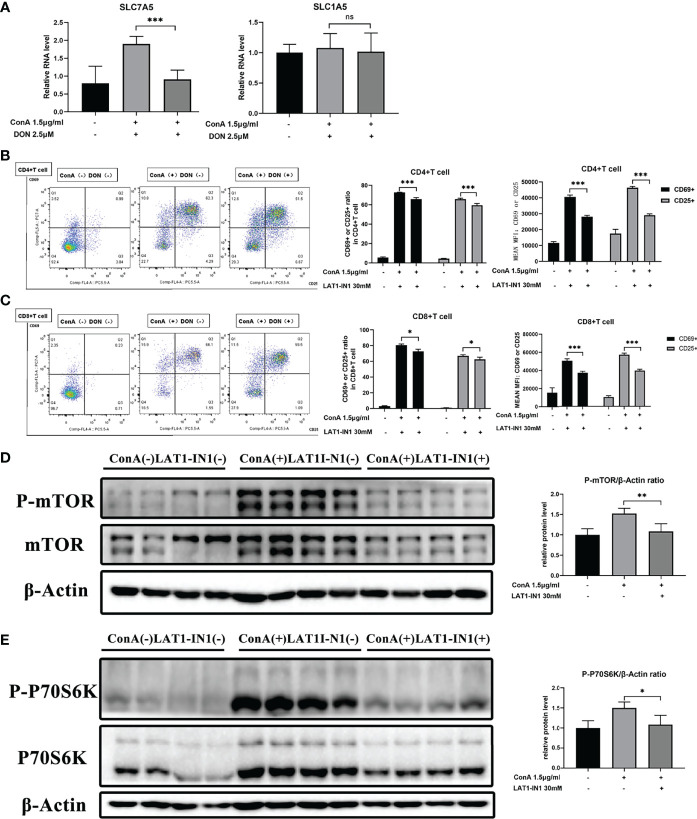
The function of DON to mTOR signaling may be mediated by amino transporter amino transporter SLC7A5. **(A)** qRT-PCR was applied to analyze amino acid transporters SLC7A5 and SLC1A5 mRNA levels. **(B, C)** Flow cytometry was applied to analyze the degree of activation (expression of CD25 and CD69) of CD4(+) **(B)** and CD8(+) **(C)** T cells with or without treating LAT1-IN1. The levels of phosphorylated mTOR **(D)** as well as phosphorylated P70S6K **(E)** were examined by western blotting. Data were expressed as means ± SEM. *P < 0.05, **P <0.01, and ***P <0.001. ns, not significant.

The levels of amino acids are closely correlated with corresponding amino acid transporters. To explore how DON makes an impact on mTOR signaling, we focused on glutamine and leucine transporters because it was reported that the function of leucine and glutamine exchanger solute carrier family 7 member 5 (SLC7A5) and glutamine transporter solute carrier family 7 member 5 (SLC1A5) affected both intracellular amino levels and activation of mTOR signaling (35–39). We discovered that DON significantly reduced the mRNA expression of SLC7A5 but had no effect on SLC1A5 (**Figure 7A**). These results suggested that SLC7A5 may play a critical part in the process of GLS inhibition to the suppression of activation of mTOR signaling. To further confirm the function of SLC7A5, we inhibited it with LAT1-IN1 (30mM) (40), the competitive antagonist of SLC7A5. After LAT1-IN1 treatment, the activation of CD4(+) and CD8(+) T cells, evidenced by the expressions of CD25 and CD69, was significantly weakened (**Figures 7B, C**). In addition, the expression levels of phosphorylated mTOR and P70S6K were inhibited by LAT1-IN1 (**Figures 7D, E**). Therefore, our findings demonstrated that GLS blocking not only suppresses T cell activation and differentiation, but also decreases mRNA expression of glutamine and leucine exchanger SLC7A5, reducing intracellular leucine level and further inhibiting activation of mTOR signaling.

## Discussions

Recent studies connecting the fields of amino acid metabolism and immunology have dramatically improved our understanding of how immune cells benefit from a metabolic reprogramming to support their activation and differentiation (41–45). The glutaminase antagonist JHU083, a prodrug of DON, has been reported to be a potential anti-inflammatory modulator to reverse experimental autoimmune encephalomyelitis through inhibiting T cells proliferation and activation (46). Another study demonstrated that GLS may have an impact on Th1 and Th17 cells differentiation (15). Emerging evidence proposed that amino acid transporters are also critical in T cell activation and differentiation (44, 45, 47). The amino acid transporter SLC7A5 is a bidirectional transporter that regulates the simultaneous transport of leucine into cells and efflux of glutamine out of cells (34) and is crucial for the activation of mTOR signalling (44, 48, 49). Notably, mTOR signaling can modulate T cells activation and differentiation (15, 48–51). Thus, we proposed a hypothesis that targeting glutamine metabolism may affect the activation and differentiation of T cell through modulating SLC7A5 and inhibiting mTOR signaling. However, it remains unknown whether targeting glutamine metabolism exerts anti-inflammatory effects on the modulation of AIH.

In this study, we evaluated the therapeutic effect of GLS antagonist JHU083 in ConA-induced AIH mice model, which was caused by a T cell-mediated autoimmune response (29). We observed a marked improvement of disease severity after treatment with JHU083. In addition, we found JHU083 or DON attenuated CD4(+) and CD8(+) T cells activation, inhibited Th1/Th17 cells and CTL differentiation *in vivo* and *in vitro*. However, the reduction of activation and differentiation of T cells may not be enough to explain the attenuation of serum transaminase and pathologic changes of livers. Blocking glutamine metabolism may also ameliorate the activation of mTOR through other mechanisms such as suppressing the production of glutathione (15) or polyamines (51), or other undiscovered mechanisms. GLS antagonist may attenuate AIH *via* other etiologies such as modulating innate immune cells as it is reported that glutamine metabolism also plays a role in innate immune cells such as macrophages (52–54). Mechanistic studies revealed that blocking glutamine metabolism lowered the mRNA expression of amino acid transporter SLC7A5, and the inhibition of SLC7A5 by LAT1-IN1 reduced the expressions of phosphorylated proteins in mTOR signaling and decreased activation of T cells. Thus, these studies showed that decreased expression of SLC7A5 mRNA, mitigated mTOR signaling and reduced activation rate of T cells and subsided Th1/Th17 cells and CTLs differentiation might be responsible for protective effect of JHU083 in AIH. We showed for the first time that JHU083 conferred protection against AIH and could be developed as novel therapeutic agent for the treatment of AIH in clinical medicine.

T cells activate and differentiate into functional subgroups need energy and metabolites, which were brought by metabolic reprogramming (51). GLS converts glutamine to glutamate to support the tricarboxylic acid cycle and redox and epigenetic reactions (15). Many researches proves that GLS antagonist inhibits the activation of T cells (15, 51, 55, 56). But it remains unclear whether it works in T cell-mediated disease models. As far as we know, our study is the first study to explore the effects of blocking glutamine metabolism on a ConA-induced T cell-mediated AIH mice model.

Then, we tried to find out how GLS antagonist ameliorated the inflammation and mitigated the necrosis and apoptosis of liver cells of AIH. Thus, we examined the differentiation of Th1/Th17 cells and CTL upon ConA stimulation *in vivo* and *in vitro*. One study demonstrated that GLS promotes differentiation and function of Th17 cells yet restrains Th1 cells *in vitro* (15, 17), but our results suggested that GLS antagonist significantly suppresses the differentiation of Th1/Th17 cells and CTL. Due to the fact that mTOR signaling is also strongly correlated with the activation and differentiation of T cells (15, 47–50), we tested the levels of the phosphorylated proteins in mTOR signaling and found that glutamine metabolism blocking significantly reduced the levels of phosphorylated mTOR and P70S6K. Other studies also demonstrated that GLS inhibition may inhibit the activation of mTOR signaling and differentiation of T cells through suppressing the production of polyamines (51) or glutathione (15) or downregulating Th17-promoting transcription factor, inducible cAMP early repressor (17). However, we proved that targeting glutamine metabolism may regulate the activation of mTOR signaling and inhibit the leucine-glutamine exchanger and increasing the intracellular level of leucine, as it is widely accepted that intracellular leucine level determines the activation of T cells (57–60).

Although the mice model of ConA-induced hepatitis is a well-recognized and typical tool to study AIH (9), it is acute and usually disappears after 48h and could only partially mimic the pathogenesis of AIH in the human body (61, 62). The present study was performed using mice and cellular experiments, and additional studies on human AIH are needed to verify the protective effects of blocking glutamine metabolism in the future.

Previous evidences show that blocking glutamine metabolism significantly impedes the proliferation of T cells *in vivo* (45) and *in vitro* (20, 51). Thus, GLS antagonist may be a potential drug to attenuate AIH in the human body, as human AIH is an immunoinflammatory chronic liver disease (63) and GLS inhibition may even protect liver from the damages caused by T cells. As AIH is a liver disease characterized by a T cell-mediated autoimmune response, we are provoked to investigate the effect of glutamine metabolism blocking on other T cell-mediated diseases, such as liver allograft rejection. Liver allograft rejection is caused by the recognition of non-self-donor alloantigens by recipient T cells and remains a significant cause of morbidity and graft failure in liver transplant recipients (64). The main treatment of liver allograft rejection is anti-rejection drugs such as glucocorticoids, tacrolimus, which have strong side effects (65). As we mentioned above, JHU083 is a prodrug of DON with weak side effects, and may be a promising drug to prevent or ameliorate the progression of liver allograft rejection.

In conclusion, we have demonstrated that GLS blocking reduces the activation and differentiation of T cells and disease severity in ConA-induced AIH mice, *via* reducing the mRNA expression of SLC7A5, lowering the level of intracellular leucine and further inhibiting the activation of mTOR signaling.

## Data Availability Statement

The original contributions presented in the study are included in the article/**Supplementary Material**. Further inquiries can be directed to the corresponding authors.

## Ethics Statement

The animal study was reviewed and approved by the Institutional Animal Care and Use Committee of State Key Laboratory of Drug Research, Chinese Academy of Sciences (Shanghai, China).

## Author Contributions

Conception and study design QY, HT, XY, YZ, HJ and QX. Acquisition of data QY, HT, XY, CP, CD, WY, WW and XG. Data analysis QY, HT, XY and CP. Data interpretation JL, HY, YZ, HJ and QX. Manuscript drafting and revising QY, HT, XY, CP, CD, WY, WW, XG, JL, HY, YZ, HJ and QX). All authors contributed to the article and approved the submitted version.

## Funding

This project was funded by National Key R&D Program of China 2018YFA0108200, National Natural Science Foundation of China grants 32000525, ‘Three-Year Action Plan for Promoting Clinical Skills and Clinical Innovation in Municipal Hospitals’ Key Supporting Projects SHDC2020CR5012 and Major clinical research projects SHDC2020CR2003A.

## Conflict of Interest

The authors declare that the research was conducted in the absence of any commercial or financial relationships that could be construed as a potential conflict of interest.

## Publisher’s Note

All claims expressed in this article are solely those of the authors and do not necessarily represent those of their affiliated organizations, or those of the publisher, the editors and the reviewers. Any product that may be evaluated in this article, or claim that may be made by its manufacturer, is not guaranteed or endorsed by the publisher.
