# Supplementary material for: Targeting Glutamine Metabolism Ameliorates Autoimmune Hepatitis via Inhibiting T Cell Activation and Differentiation
Source: Front Immunol. 2022 May 19;13:880262. doi: 10.3389/fimmu.2022.880262 (PMC9160195; doi:10.3389/fimmu.2022.880262)
Supplement: Supplementary file 1 [file DataSheet_1.docx]

**Supplementary Figures**


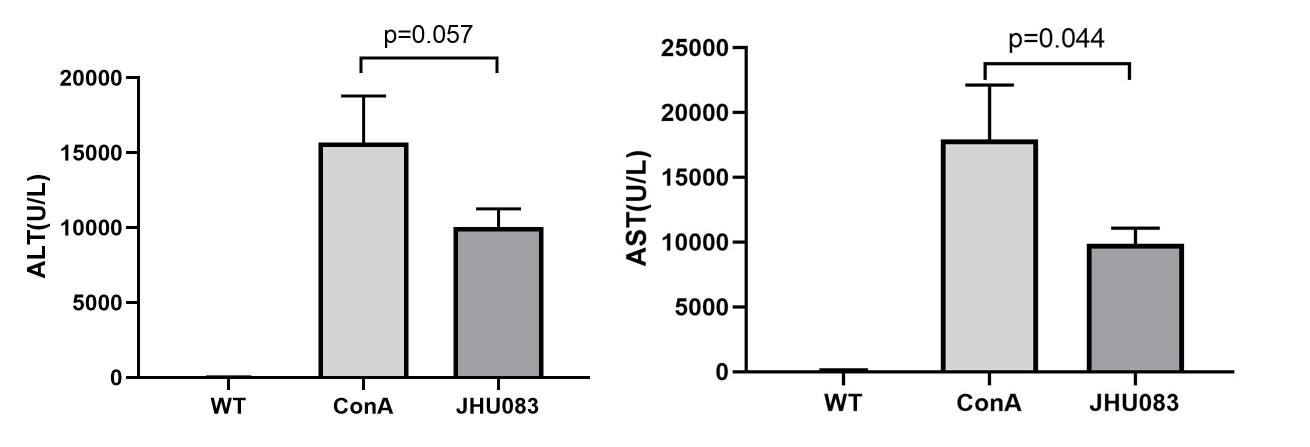


Supplementary Figure 1. The effect of JHU083 on the plasma ALS and AST of ConA (20mg/kg) induced AIH mice.


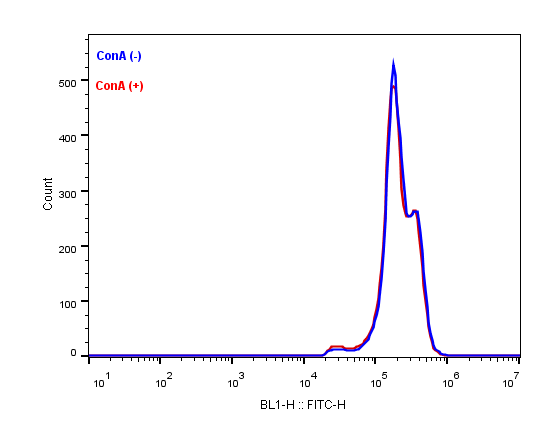


Supplementary Figure 2. Proliferation status of transplanted splenocytes (CFSE labeled) after ConA induction for 6h in vivo.


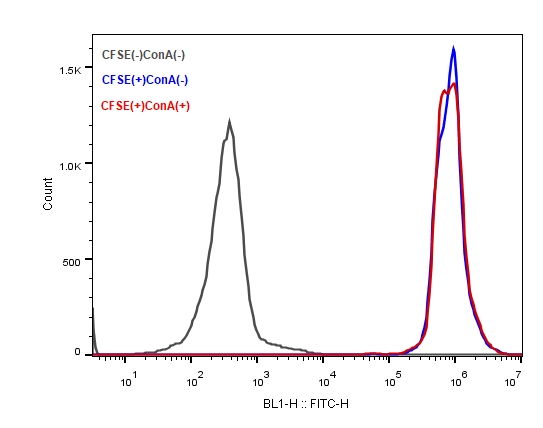


Supplementary Figure 3. Proliferation status of splenocytes with or without ConA stimulation for 24h in vitro.


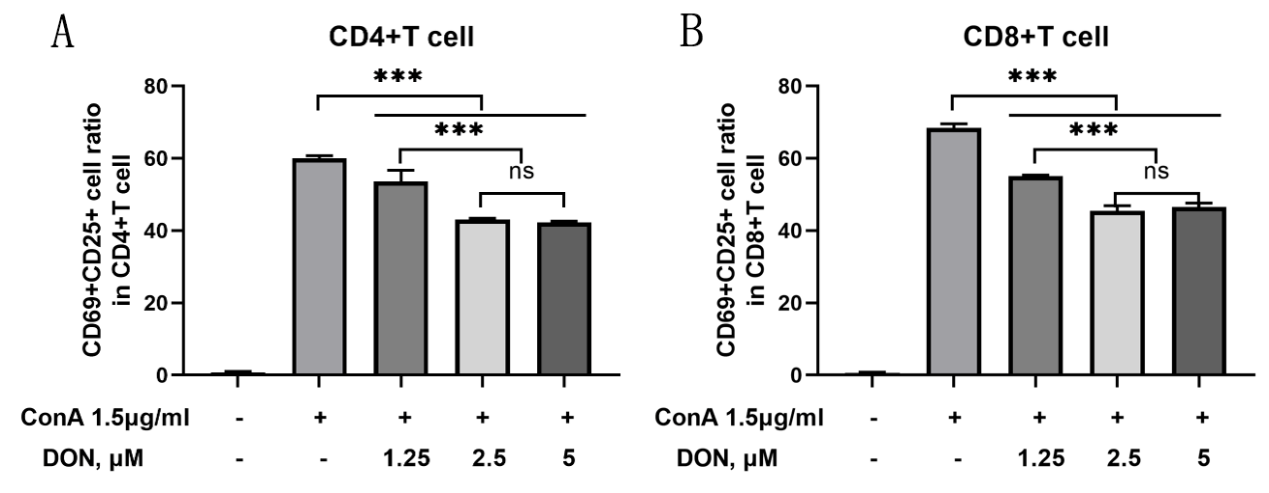


Supplementary Figure 4. The influence of different concentration of DON on the activation of CD4+ and CD8+ T cells. A: CD4+T cells. B: CD8+T cells.


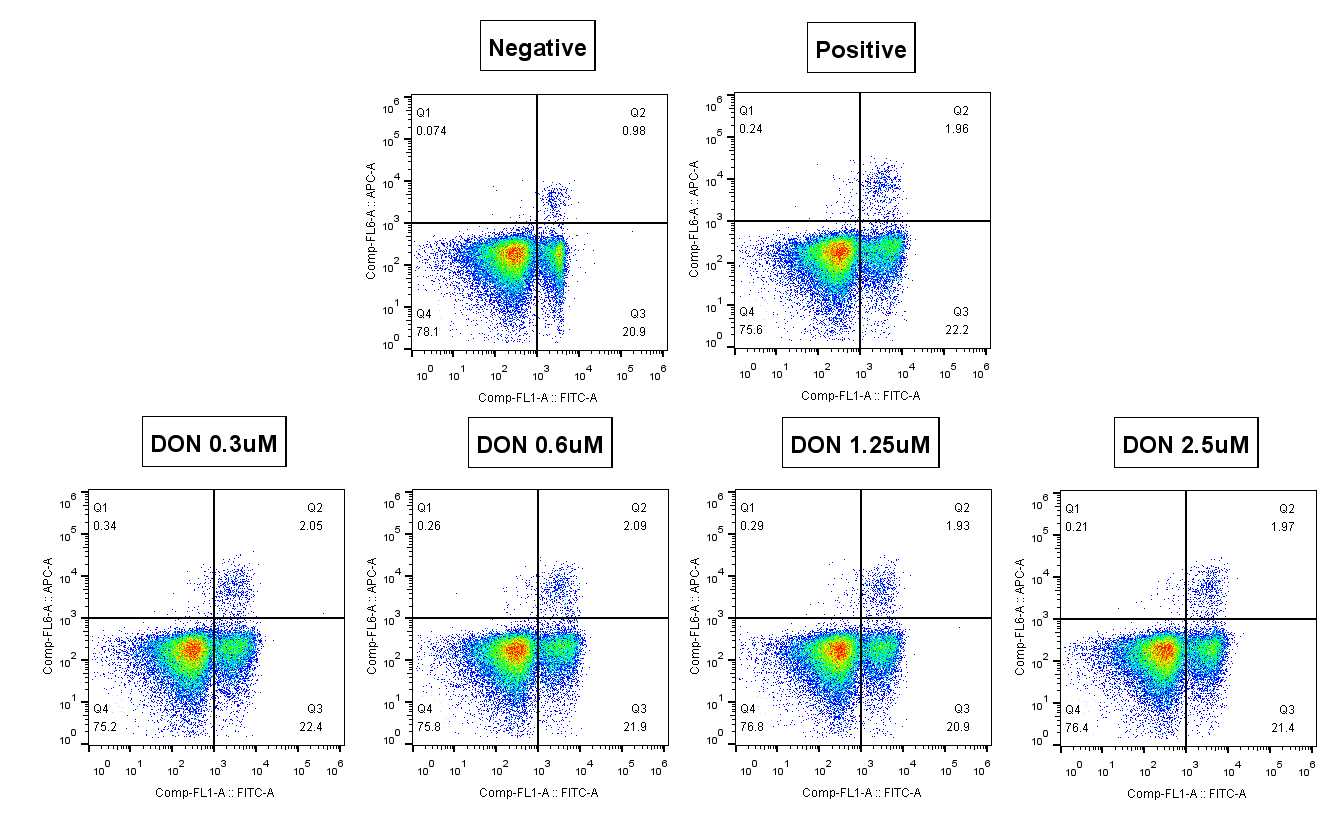


Supplementary Figure 5. Proportion of Treg cells after ConA induction and different concentration of DON inhibition.
